# Functionalized Fullerene Potentially Inhibits SARS-CoV-2 Infection by Modulating Spike Protein Conformational Changes

**DOI:** 10.3390/ijms241914471

**Published:** 2023-09-23

**Authors:** Kaifeng Liu, Fangfang Guo, Yingying Ma, Xiangyu Yu, Xueqi Fu, Wannan Li, Weiwei Han

**Affiliations:** 1 Key Laboratory for Molecular Enzymology and Engineering of Ministry of Education, School of Life Sciences, Jilin University, Changchun 130012, China; liukf1220@jlu.edu.cn (K.L.); mayy2920@mails.jlu.edu.cn (Y.M.); 2 Edmond H. Fischer Signal Transduction Laboratory, School of Life Sciences, Jilin University, Changchun 130012, China; guoff15@mails.jlu.edu.cn (F.G.); yuxy1321@mails.jlu.edu.cn (X.Y.); fuxq@jlu.edu.cn (X.F.)

**Keywords:** molecular dynamics simulation, SARS-CoV-2, sulfated polysaccharide, functionalized fullerene, Markov state model

## Abstract

The disease of SARS-CoV-2 has caused considerable morbidity and mortality globally. Spike proteins on the surface of SARS-CoV-2 allow it to bind with human cells, leading to infection. Fullerenes and their derivatives are promising SARS-CoV-2 inhibitors and drug-delivery vehicles. In this study, Gaussian accelerated molecular dynamics simulations and the Markov state model were employed to delve into the inhibitory mechanism of Fullerene–linear-polyglycerol-b-amine sulfate (F–LGPS) on spike proteins. During the study, it was discovered that fullerene derivatives can operate at the interface of the receptor-binding domain (RBD) and the N-terminal domain (NTD), keeping structural domains in a downward conformation. It was also observed that F-LGPS demonstrated superior inhibitory effects on the XBB variant in comparison to the wild-type variant. This study yielded invaluable insights for the potential development of efficient therapeutics targeting the spike protein of SARS-CoV-2.

## 1. Introduction

The disease of COVID-19 has had a significant impact on the economy and society globally, resulting in deaths, healthcare shortages, reduced economic activity, social isolation, and mental-health deterioration. It is caused by the severe acute respiratory syndrome coronavirus 2 (SARS-CoV-2) [1,2,3]. The XBB strain is a highly transmissible and immune-escaping variant of the Omicron variant, which requires attention to vulnerable populations and close monitoring [4].

According to previous research, the first step in infection is the binding of the spike (S) protein to ACE2 [5]. The homotrimeric S protein is located on the surface of the viral membrane, and each monomer consists of two domains, S1 and S2, which are responsible for receptor binding and membrane fusion, respectively. The S1 domain is composed of the receptor-binding domain (RBD) and the N-terminal domain (NTD) [6]. The binding affinity of ACE2 varies, depending on the orientation of the RBD. The closed state in which all RBDs are downward is inaccessible to ACE2 and, thus, it is inactive, while an open state with RBDs flipped upward can bind to the receptor [7,8].

Specific sulfated polysaccharides, including fucoidans and heparin, can bind tightly to the S protein of SARS-CoV-2 in vitro, which suggests that they can act as decoys to interfere with S-protein binding to the heparan-sulfate co-receptor in host tissues, inhibiting viral infection [9]. Functionalized fullerene can be used as a drug-delivery agent with inhibitory activity [10,11], and animal experiments have also proven the good pulmonary-drug-delivery effect of fullerenes [12]. Fullerene-linear polyglycerol b-amine sulfate (F–LPGS) is a kind of functionalized fullerene that can inhibit SARS-CoV-2. It displayed an IC50 of 0.32 × 10^−3^ M against the wild-type variant, and IC_50_ between 2.02 × 10^−3^ and 0.20 × 10^−3^ M against the SARS-CoV-2 Omicron BA.5 variant. Fullerene derivatives have shown potential in inhibiting SARS-CoV-2 [13].

The Gaussian accelerated molecular dynamics simulation (GaMD) [14] is a useful method to investigate the conformational changes of proteins, protein folding, protein–ligand binding, etc. [15,16,17,18,19]. In the GaMD approach, the harmonic boost potential was added so that the energy barrier could be reduced by smoothing the potential surface and, thus, accelerating the transition between different conformational states for the purpose of enhanced sampling. Here, the increased lifting potential followed the Gaussian distribution, allowing the original potential surface to be easily recovered. Thus, this enhanced simulation approach is very suitable for studying the dynamics of complex biological systems.

Markov state models [20] can cluster protein conformations in molecular dynamics trajectories and separate them into classes called microstates. Each microstate corresponds to a state in the Markov model state’s space, and there is a transition probability between each state, forming a transition-probability matrix. By using the transfer-probability matrix and flux-analysis methods, the dynamic relationships between various microstates can be analyzed.

In this study, we employed Gaussian accelerated molecular dynamics (GaMD) simulations and the Markov state model to investigate the inhibitory mechanism of F-LGPS on the spike protein, especially towards RBD and NTD, in both the wild-type variant and the Omicron variant of SARS-CoV-2. 

## 2. Results

### 2.1. Structural Stability and Dynamics Properties of the Four Systems

**Figure 1:**
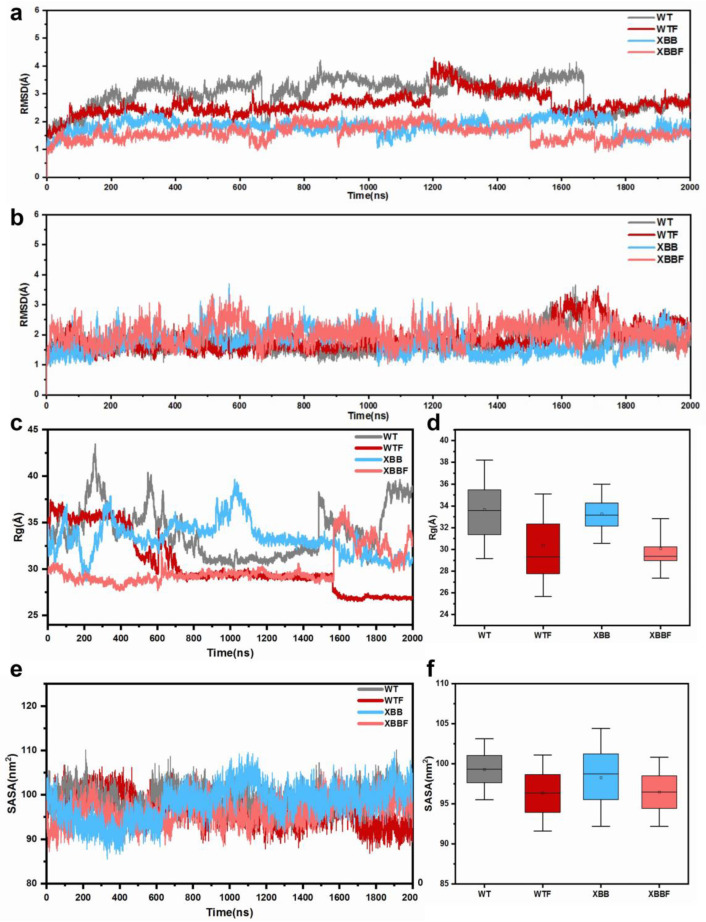
(**a**) The temporal evolution of the RMSDs of NTD. (**b**) The temporal evolution of the RMSDs of RBD. (**c**) The temporal evolution of the Rg values. (**d**) Average Rg values. (**e**) The temporal evolution of the SASA values of RBD. (**f**) Average SASA values.

The AMBER 16 software was used to perform 2000 ns GaMD simulations at 300 K for WT, WTF(WT–F–LPGS), XBB, and XBBF(XBB–F–LPGS) systems, respectively. To evaluate the stability of the simulations, the root-mean-square deviation (RMSD) of the Cα atoms was calculated; the RMSD values of the NTD and RBD are shown in Figure 1a,b, respectively. The RMSD of NTD in both WT and WTF groups was higher compared to XBB and XBBF, indicating a slightly lower stability compared to the XBB and more conformational changes. The RMSD values of the RBD in all four groups were similar. Overall, the RMSD of all four systems was within 4 Å, suggesting the good overall stability of the simulation systems and their suitability for further analysis.

**Figure 2:**
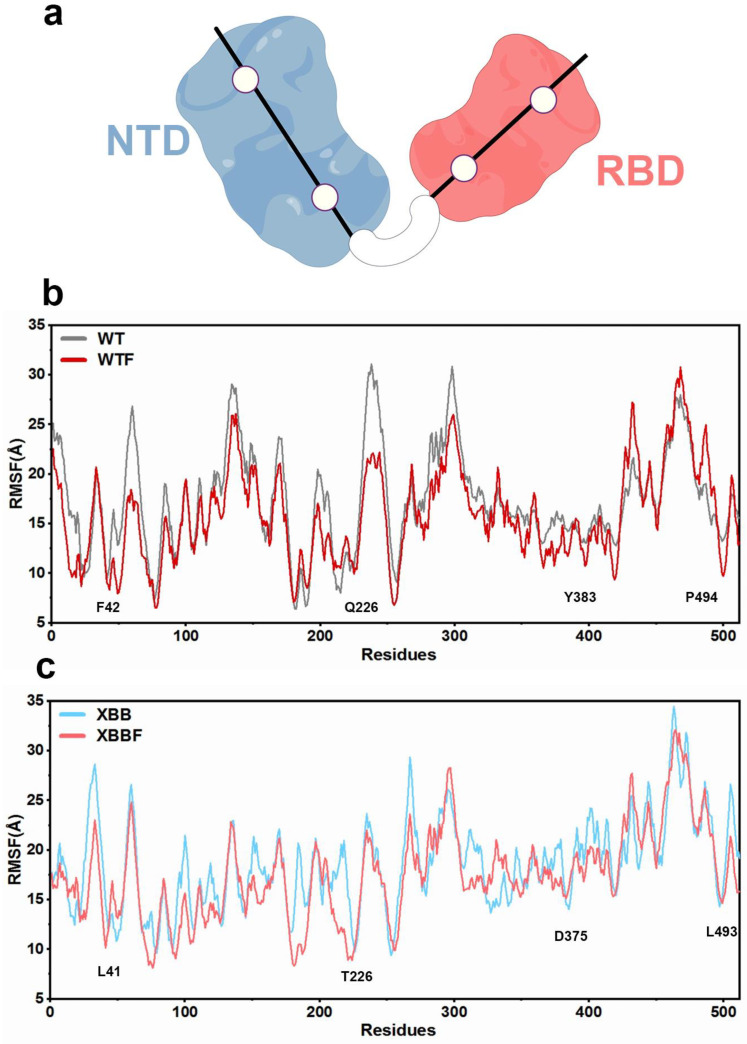
(**a**) A simplified representation of the residues along the central axis of the N-terminal domain (NTD) and receptor-binding domain (RBD). (**b**) The RMSFs of the Cα atoms in the WT and WTF systems. (**c**) The RMSFs of Cα atoms in the XBB and XBBF systems (by Figdraw).

The radius-of-gyration value is shown in Figure 1c, and the mean value is presented in Figure 1d. After binding with F–LPGS, the overall Rg of both the WTF and the XBBF decreased, indicating a closed conformation after binding, and the protein was not in an extended conformation. The SASA of the RBD also decreased, as shown in Figure 1e,f, indicating a smaller hydrophilic area, which suggested that the protein was in a closed conformation, with a reduced surface area. We then calculated the RMSF values of the Cα atoms for the four systems to define stable residues. By selecting the centroid of the most stable residues with the lowest RMSF value located on the axis of RBD and NTD domains, we defined dihedral angles that described the relative motion of the RBD and NTD. The results of the RMSF and the chosen residues are shown in Figure 2. 

**Figure 3:**
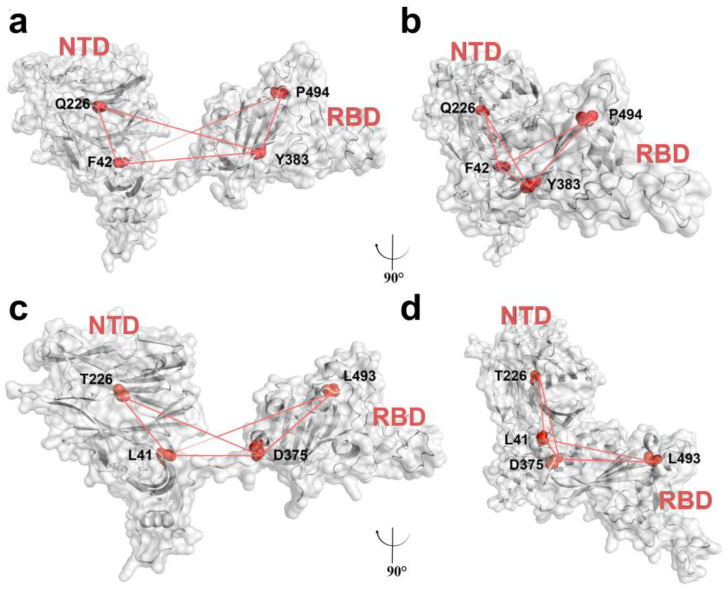
(**a**) Frontal view of the selection of four residues and their dihedral angles in the WT system. (**b**) Side view of the WT system. (**c**) Frontal view of the selection of four residues and their dihedral angles in the XBB system. (**d**) Side view of the XBB system.

The defined dihedral angles were Q226-F42-Y383-P494 for WT and WTF, and T226-L41-D375-L493 for XBB and XBBF. Frontal views and side views of the selection of residues and their dihedral angles in the WT and XBB systems are displayed in Figure 3.

**Figure 4:**
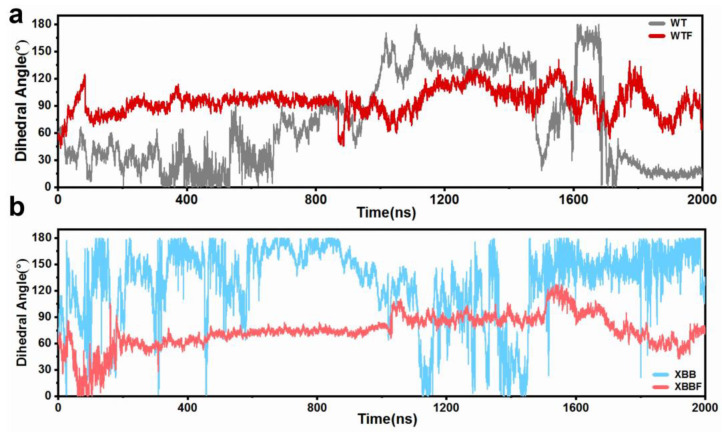
(**a**) Variation of defined dihedral angles over 2000 ns for the WT and WTF systems. (**b**) Variation of defined dihedral angles over 2000 ns for the XBB and XBBF systems.

**Figure 5:**
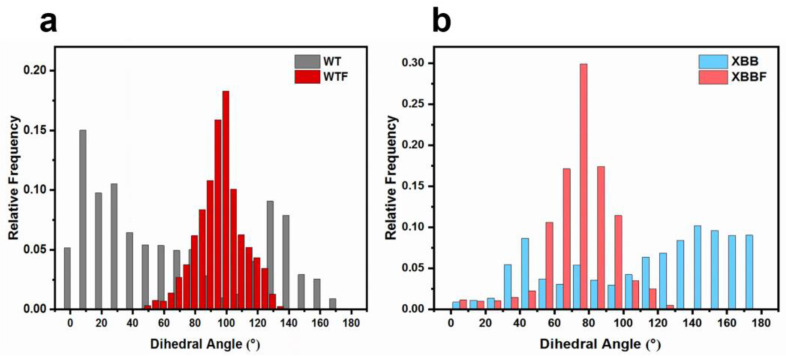
(**a**) Dihedral angles’ relative frequency distribution for the WT and WTF systems. (**b**) Dihedral angles’ relative frequency distribution for the XBB and XBBF systems.

We analyzed the changes in the dihedral angle over 2000 ns. The changes in the dihedral angles are shown in Figure 4, and the frequency distribution is presented in Figure 5. When combined with the F–LPGS, the fluctuations in the angle changes were smaller, the angle distribution became more concentrated, and the RBD and NTD planes maintained near-vertical positions, resulting in a more stable closed conformation. These results were observed in both the WTF and the XBBF system.

### 2.2. Principle Component Analysis and Markov State Model

**Figure 6:**
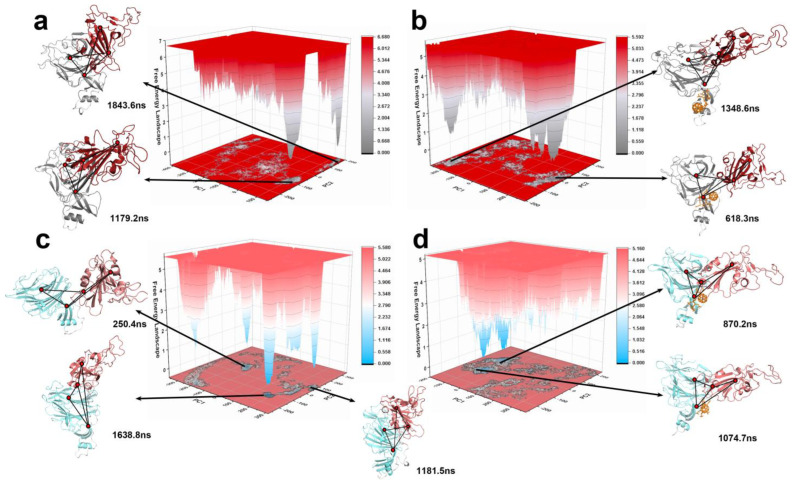
Free-energy landscapes of the four systems and the conformations corresponding to the lowest energy. (**a**) WT system. (**b**) WTF system. (**c**) XBB system. (**d**) XBBF system.

A PCA analysis of all the Cα atoms, performed for the four systems, is shown in Figure 6. The PC1 and PC2 accounted for more than 70%, as shown in Table 1, reflecting the reliability of the results. The free-energy landscape was constructed using the PCA for four systems, identifying low-energy stable representative conformations. In the WTF and XBBF systems, the conformations in the energy wells were all downward conformations, while the low-energy conformations obtained from the energy wells of the apo protein were extended upward conformations, along with a small number of metastable states between the upward and downward conformations.

**Figure 7:**
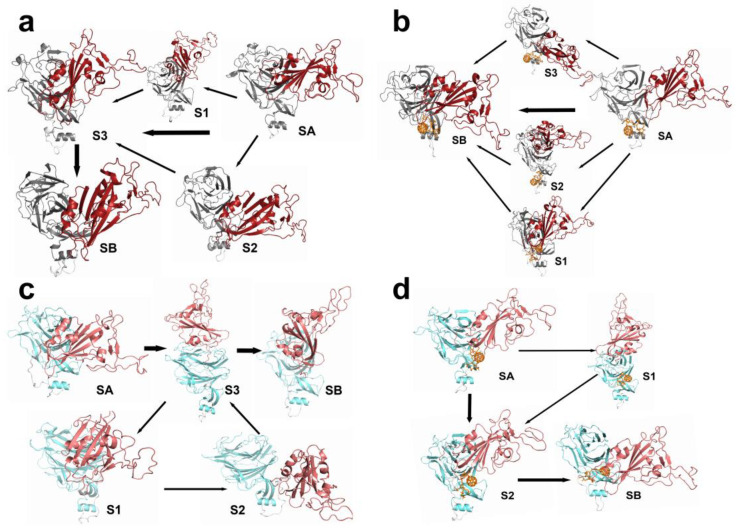
Conformational transition pathways between different states in the four systems. (**a**) WT system. (**b**) WTF system. (**c**) XBB system. (**d**) XBBF system.

To construct a Markov state model (MSM), performed simulations to generate a trajectory of conformations over time. Next, the backbone-torsion angles were extracted from the trajectories to characterize the conformational space. To simplify the conformational space, a time-lagged independent component analysis (TICA) was applied to reduce the dimensions. Subsequently, the reduced-dimension data were clustered into discrete states using k-means clustering algorithms. The VAMP2 scores for different numbers of cluster centers are shown in Figure S1, and the results of the clustering are shown in Figure S2. The lag time was determined to be 0.8 ns, as shown in Figure S3. The transition probabilities between the states were estimated by constructing a transition matrix, which captured the dynamics of the system. The MSM was then validated by Chapman–Kolmogorov tests (Figure S4), ensuring its reliability. The MSM described the transition of the states, as shown in Figure 7. The total flux from the initial state (SA) to the target state (SB) was decomposed into pathways, which can be seen in Table 2.

In the systems without F–LPGS, the protein tended to stabilize in a relatively open conformation after the RBD wobble compared to the systems with F–LPGS. Th WT was stable in a semi-open conformation, while the XBB was more open in a stable conformation than the WT, and the RBD was more flexible and easier to bind to the ACE2. This partly explained its high infectivity. In the WTF and XBBF systems, the protein directly entered the closed conformation at a high flux from the initial state, and a small portion transitioned to the closed conformation after undergoing swinging and a small number of open conformations.

### 2.3. Analysis of the Interaction between Protein and Ligand

**Figure 8:**
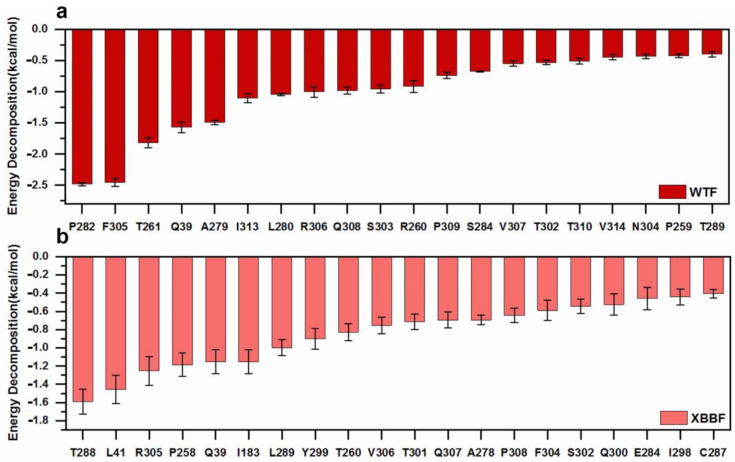
(**a**) The 20 residues contributing the most to the free energy in the binding of WT and F–LPGS. (**b**) The 20 residues contributing the most to the free energy in the binding of XBB and F–LPGS.

The results of the MM-PBSA are shown in Table 3. The binding free energy of the WT–F–LPGS was −41.05 ± 0.59 KJ/mol, while the binding free energy of the XBB–F–LPGS was −45.37 ± 1.36 KJ/mol; the binding free energy was lower in the XBB–F–LPGS, indicating a better affinity. Figure 8 shows the binding-energy contributions of the 20 highest-ranked residues in the WTF and XBBF systems. The residues in the XBB provided greater binding energy than those in the WT. 

**Figure 9:**
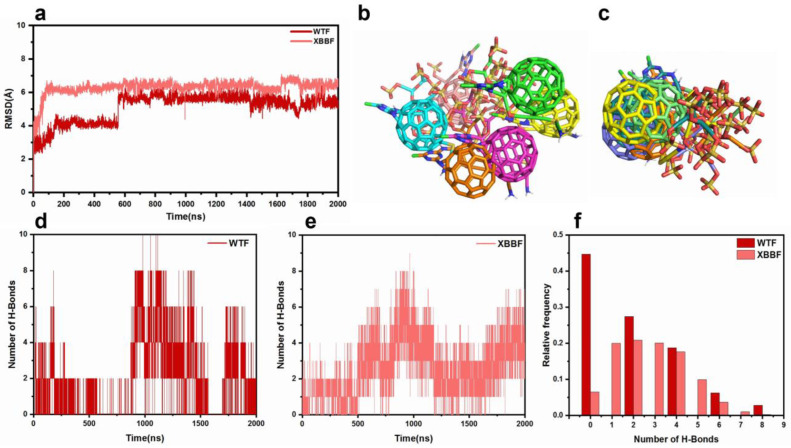
(**a**) RMSD of F-LGPS in the WTF and XBBF systems. (**b**) Ligand poses of 10 superimposed structures over 2 μs for WTF. (**c**) Ligand poses of 10 superimposed structures over 2 μs for XBBF. (**d**) Numbers of hydrogen bonds between receptor and ligand in the WTF system. (**e**) Numbers of hydrogen bonds between receptor and ligand in the XBBF system. (**f**) Frequency distribution of hydrogen bond numbers.

We also calculated the RMSD of the ligands and performed clustering and hydrogen-bond analyses, as shown in Figure 9. The analysis of the ligand showed that the binding position of the ligand in the XBB system was more stable, and more hydrogen bonds were formed in the XBBF system during the 2000-nanosecond simulation. The F–LPGS showed better inhibition of the XBB variant than the WT.

## 3. Discussion

In our study, we employed Gaussian accelerated molecular dynamics (GaMD) simulations and Markov state models to investigate the inhibitory mechanism of F-LGPS on the spike protein, particularly for the RBD and NTD domains, in both the wild-type variant and the Omicron (XBB) variant of SARS-CoV-2. Our findings reveal that fullerene derivatives, such as F-LGPS, can effectively bind at the RBD–NTD interface, maintain the structural domains in a closed conformation, and reduce the likelihood of transitioning to an open or upward conformation. This action inhibits SARS-CoV-2 infection, providing valuable insights for the development of novel SARS-CoV-2 inhibitors.

Our study also demonstrates that these fullerene derivatives exhibit a stronger inhibitory effect on the XBB (Omicron) variant compared to the wild-type. This observation suggests the potential applicability of these derivatives in suppressing the spread of emerging SARS-CoV-2 variants, such as the highly infectious Omicron variant. The binding-free-energy analysis and hydrogen-bond analysis further support the enhanced inhibitory effect of F-LGPS on the XBB variant.

The results of the Markov state model reveal the protein’s dynamic behavior and conformational changes in the presence and absence of F-LGPS. In systems without F-LGPS, the protein tends to stabilize in a relatively open conformation after RBD wobbling compared to systems with F-LGPS. In the presence of F-LGPS, the protein directly enters the closed conformation at a high level of flux from the initial state, and a small portion transitions into a closed conformation after undergoing swinging and a small number of open conformations.

These findings have important implications for the ongoing battle against the COVID-19 pandemic and its evolving variants. By understanding the inhibitory mechanism of fullerene derivatives on the spike protein, we can contribute to the development of novel SARS-CoV-2 inhibitors with enhanced efficacy and safety profiles. Future studies should focus on the experimental validation of these findings and the optimization of fullerene derivatives for improved efficacy and safety in clinical applications. Additionally, further research into the inhibitory effects of fullerene derivatives on other emerging SARS-CoV-2 variants will be crucial in addressing the ongoing challenges posed by the COVID-19 pandemic.

## 4. Materials and Methods

### 4.1. System Preparation

**Figure 10:**
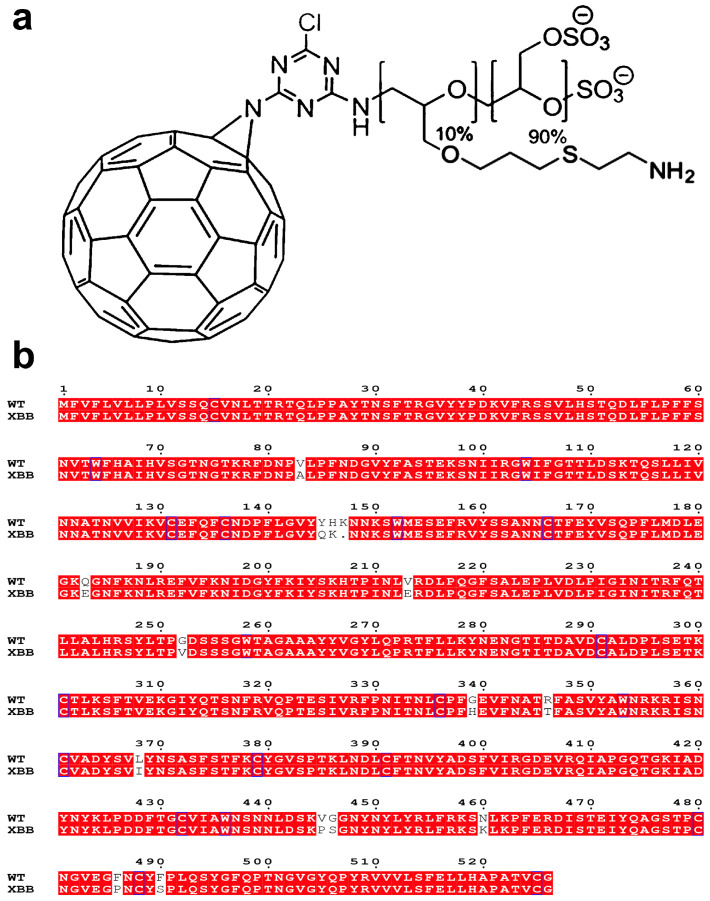
(**a**) The structure of F–LPGS. (**b**) The sequences of 1-526 in spike for WT and XBB 1.5.

The 2D structure of fullerene–linear-polyglycerol-b-amine-sulfate (F–LPGS) is shown in Figure 10a, its 3D structure was built using Avogadro software [21], and we retained five structural units for it. Gaussian 09 [22] was used to optimize the structure at the level of B3LYP/6-31G* to obtain the optimal conformation of F–LPGS for molecular docking. The NTD and RBD of wild-type variant (WT) consisted of residues 14–526 in chain A of the SARS-CoV-2 spike protein (PDB code: 6VXX) [23]; since several fragments were not resolved in these Cryo-EM structures, SWISS-MODEL [24,25,26] was employed to replenish the missing atoms. Next, we constructed the 3D structure of NTD and RBD (residues 14–525) of XBB.1.5 [27] using Alphafold2 Colab [28,29]. The sequences of WT 1-526 and XBB 1-525 are shown in Figure 10b. 

**Figure 11:**
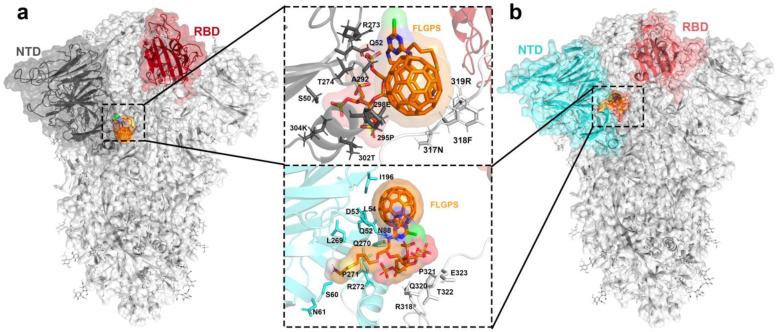
(**a**) Binding pose for WT–F–LPGS complex. (**b**) Binding pose for XBB–F–LPGS complex. Residues that interacted with the ligands are shown as sticks.

The F–LPGS was docked into the position between the RBD and NTD domains with Autodock Vina 1.2.0 [30,31] to form the WT–F–LPGS (WTF) and XBB–F–LPGS (XBBF) systems, respectively. The size of the docking box was set to x = 70, y = 70, and z = 70, and the spacing between grid points was set to 0.375 Å. The lowest-energy structures were selected from docking results as the initial structures for the MD simulations. Binding poses obtained from docking are illustrated in Figure 11.

### 4.2. Molecular Dynamics Simulations

Systems under study were designated as follows: WT for NTD and RBD of wild-type variant, WTF for WT–F–LPGS, XBB for NTD and RBD of XBB.1.5, and XBBF for XBB–F–LPGS. The protein residues in the systems were renumbered, with 14–526 in WT renumbered as 1–513, and 14–525 in XBB renumbered as 1–512.

The pmemd. cuda module in AMBER 16 (University of California, San Francisco, CA, USA) [32] was used to perform conventional MD simulations for four model systems. The force fields ff14SB [33], GAFF2 [34], and TIP3P [35] in Amber16 were employed in Leap module to parameterize the proteins, F–LPGS, and water molecules, respectively. Subsequently, each system was dissolved in an octahedral box using the TIP3P water model. The distance between the solute surface and the box was set to 12 Å. To prevent edge effects, periodic boundary conditions (PBCs) were applied to the three systems. Appropriate amounts of Na+ ions were added to neutralize the system. All bonds involving hydrogen atoms were constrained using the SHAKE algorithm [36]. The particle-mesh Ewald (PME) algorithm [37] was used to handle non-bonded electrostatic interactions, and the cut-off was set to 8 Å. Before the production simulation, energy minimization was executed for the four systems to eliminate atomic collisions in the initial structure. In the minimization phase, the steepest-descent algorithm and conjugate-gradient algorithm were performed for 500 steps each. Next, the four systems were gradually heated from 0 K to 300 K in NVT ensemble at 50 ps. Finally, 50 ns simulations were carried out for the equilibrium of the systems under the NPT ensemble. The entire simulation used a time step of 2 fs, and a Langevin thermostat [38] with a collision frequency of 1 ps.

The initial structures used by the GaMD simulations were obtained from the well-balanced structure of the cMD simulations. In this study, we applied the dual-potential boost to the GaMD simulations. Boosting was applied to both the total and dihedral potential energy (igamd = 3). The dual-potential-boost parameters were defined by the previous 50 ns cMD simulations. Subsequently, multiple replicates of GaMD simulations were carried out for four systems, with coordinates saved every 10 ps, and the total simulation time for each system was 2000 ns. The GaMD trajectories were used for subsequent analysis.

### 4.3. Trajectory Analysis

Trajectory analyses, including the root-mean-square deviations (RMSD), radius of gyration (Rg), solvent-accessible surface area (SASA), root-mean-square fluctuations (RMSF), and dihedral angle, were computed using Amber16’s Cpptraj module [39]. Principal component analysis (PCA) [40] is a widely used dimensionality-reduction method that describes the coordinated motions of entire proteins, which were also calculated using Cpptraj in this study. The free-energy landscapes (FELs) are often used to find the dominant conformation and its corresponding potential barrier. Here, we used the ddtpd software by Tian Lu to construct the FEL.

### 4.4. MSM Analysis

The use of MSM analysis is a powerful tool for transforming groups of short trajectories into scientifically meaningful dynamic models. In this study, PyEMMA 2.5.7 [41] was utilized to construct MSM through the following workflows.

First, the backbone-torsion angles were extracted from each frame in the MD trajectories to discriminate different conformations. Next, the dimension of the space was reduced to two collective coordinates using time-lagged independent component analysis (TICA) [42]. This technique retains 95% of the dynamic variance of the original data and identifies the slowest modes in a feature space by maximizing the autocorrelation of reduced coordinates. The TICA method is preferred for MSM construction over principal component analysis (PCA), since it takes into account kinetic information.

We then used VAMP-2 scores, where VAMP stands for variational approach for Markov process, to ascertain the number of cluster centers [43]. Next, the conformations for each system were clustered into microstates using the k-means algorithm. A transition-count matrix was constructed by counting the number of transitions between each pair of microstates at an appropriate lag time using the sliding-window approach. The transition-probability matrix was then obtained using the Bayesian MSM estimator [44]. Timescales were examined to determine when the system becomes Markovian [45]. As shown in Figure S3, a lag time of 0.8 ns was chosen.

The Chapman–Kolmogorov test [46] was employed to evaluate the validity of the Bayesian Markov model, revealing a nearly perfect agreement between the estimated transition probabilities calculated from the MD data and the predictions of the MSMs, suggesting the validity of the MSMs. Subsequently, these microstates were further divided into macrostates using the PCCA algorithm [47]. Finally, the TPT was used to elucidate the transitions between these macrostates and the highest-flux pathway [48]. The conformations presented in Figure 9 are representative of each macrostate.

### 4.5. MM-PBSA Calculations

The accurate calculation of protein–protein binding free energy is of great importance in biological and medical science. This work used the molecular mechanics/Poisson–Boltzmann surface area (MM/PBSA) method to explore the proteins’ binding affinity to F–LPGS [49,50,51].

The binding free energy (ΔGbind) can be expressed by the following Equation.

ΔG_bind_ = ΔH − TΔS (1)

The changes in the protein and ligand upon binding were similar in all systems, with very small entropy differences; therefore, the calculation of the solvate entropy term is omitted. The enthalpy change (ΔH) was computed as the sum of changes in the gas-phase energy (ΔE_MM_) and the solvation-free energy (ΔG_sol_), averaged over a conformational ensemble generated by MD simulations:

ΔH = ΔE_MM_ + ΔG_sol_ (2)

The ΔE_MM_ was estimated using the following equation:

ΔE_MM_ = ΔE_ele_ + ΔE_vdW_ + ΔE_int_ (3)

where ΔE_ele_, ΔE_vdW_, and ΔE_int_ represent the electrostatic, vdW, and internal energies, corresponding to the bond, angle, and dihedral energies, respectively.

In this study, the conformational structures of the protein–ligand complex, protein, and ligand are regarded as a rigid body. Thus, the ΔE_int_ between the complex and the isolated components can offset each other, because this energy term was calculated from the same MD simulated trajectory. The ΔGsol was used to indicate the sum of the polar solvation-free energy (ΔG_pb_) and non-polar solvation-free energy (ΔG_np_).

ΔG_sol_ = ΔG_pb_ + ΔG_np_ (4)

The ΔG_pb_ was determined by solving the linearized Poisson–Boltzmann equation using the PBSA program in the AMBER 16 suite. Next, 500 snapshots were extracted from the final trajectory for MM/PBSA calculation.

## 5. Conclusions 

In conclusion, our study has two main findings. First, fullerene derivatives can function at the RBD–NTD interface, maintain structural domains in a closed conformation, reduce the transition to an open or upward conformation, and thus inhibit SARS-CoV-2 infection. Second, these derivatives exhibited better inhibitory effects on the XBB variant, suggesting their potential applicability in suppressing the Omicron variant. Our research may provide theoretical support for the development of novel SARS-CoV-2 inhibitors.

## Figures and Tables

**Table 1:** The proportions of the first two principal components in the four systems.

Systems	PC1	PC2
WT	54.81%	25.36%
WTF	63.58%	20.72%
XBB	65.13%	13.32%
XBBF	65.45%	7.83%

**Table 2:** The pathways obtained from TPT (transition-path theory)-based analysis of the four systems.

System	Pathways	Percentage of Total Coarse Flux (%)
WT	SA→S1→S3→SB	0.69
	SA→S3→SB	98.92
	SA→S2→S3→SB	0.39
WTF	SA→S3→SB	0.30
	SA→SB	99.01
	SA→S2→SB	0.59
	SA→S1→SB	0.10
XBB	SA→S3→SB	91.75
	SA→S1→S2→S3→SB	8.25
XBBF	SA→S1→S2→SB	3.23
	SA→S2→SB	96.77

**Table 3:** The results of MM-PBSA.

System	WTF	XBBF
ΔE_vdW_	−85.67 ± 0.57	−85.04 ± 1.71
ΔE_ele_	−73.14 ± 1.73	−61.45 ± 3.36
ΔG_gas_	−158.81 ± 1.88	−146.48 ± 3.16
ΔG_solv_	117.75 ± 1.89	101.11 ± 3.58
ΔG_total_	−41.05 ± 0.59	−45.37 ± 1.36

## Data Availability

All the data can be reasonably requested from the authors.

## Supplementary Materials

The following supporting information can be downloaded at: https://www.mdpi.com/article/10.3390/ijms241914471/s1.
