# Supplementary material for: Functionalized Fullerene Potentially Inhibits SARS-CoV-2 Infection by Modulating Spike Protein Conformational Changes
Source: Int J Mol Sci. 2023 Sep 23;24(19):14471. doi: 10.3390/ijms241914471 (PMC10572755; doi:10.3390/ijms241914471)
Supplement: Supplementary file 1 [file ijms-24-14471-s001.zip › ijms-2583449-supplementary.pdf]

Supplementary Materials for

Functionalized Fullerene Potentially Inhibits SARS-CoV-2 Infection by Modulating Spike Protein Conformational Changes

Kaifeng Liu [1,†], Fangfang Guo [2,†], Yingying Ma [1], Xiangyu Yu [2], Xueqi Fu [2], Wannan Li [2,*] and Weiwei Han [1,*]

[1] Key Laboratory for Molecular Enzymology and Engineering of Ministry of Education, School of Life Sciences, Jilin University, Changchun 130012, China; liukf1220@jlu.edu.cn (K.L.); mayy2920@mails.jlu.edu.cn (Y.M.)
[2] Edmond H. Fischer Signal Transduction Laboratory, School of Life Sciences, Jilin University, Changchun 130012, China; guoff15@mails.jlu.edu.cn (F.G.); yuxy1321@mails.jlu.edu.cn (X.Y.); fuxq@jlu.edu.cn (X.F.)
* Correspondence: liwannan@jlu.edu.cn (W.L.); weiweihan@jlu.edu.cn (W.H.)
† These authors contributed equally to this work.

This PDF file includes:

Figures S1 to S4

Figure S1

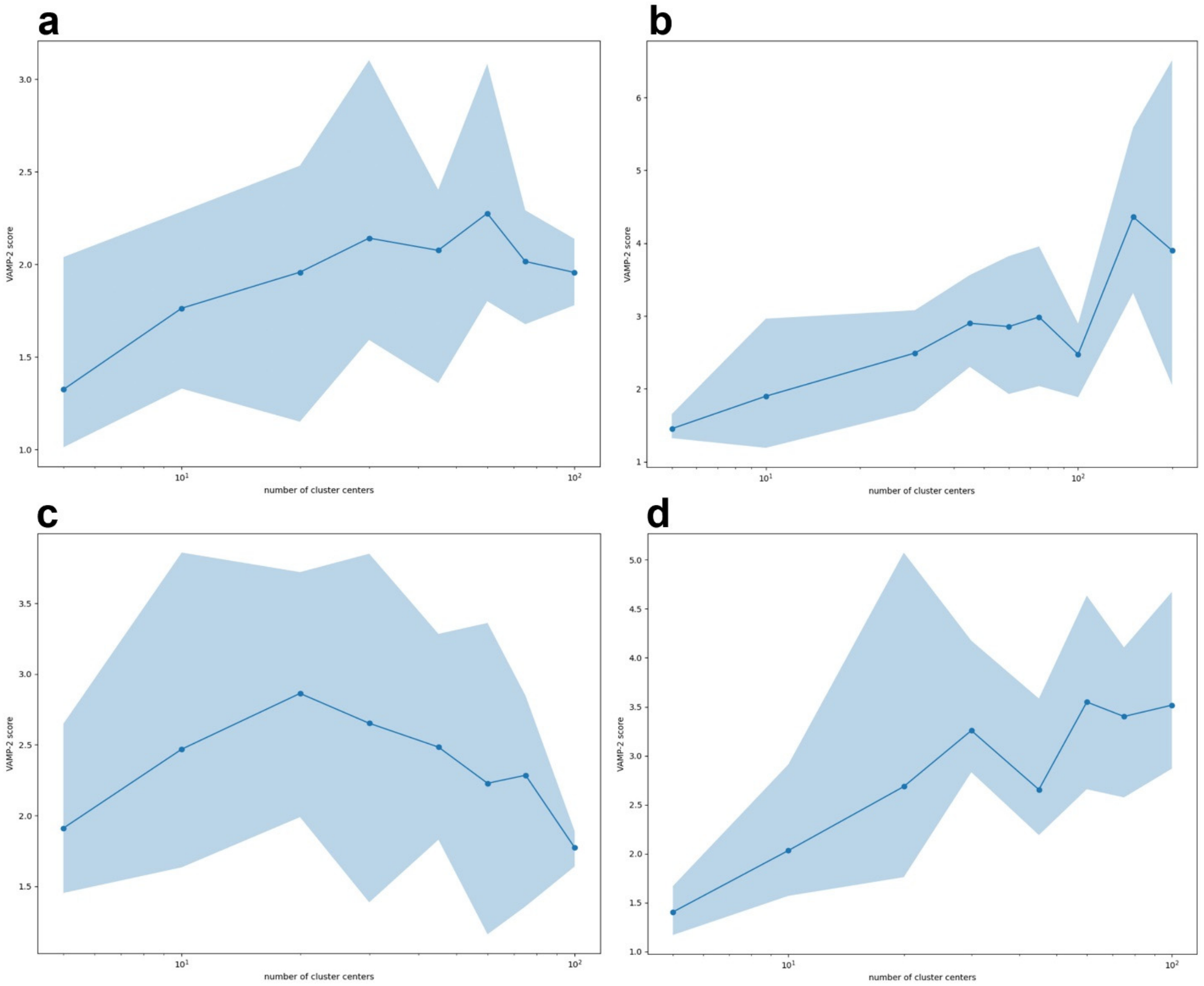

Figure S1. VAMP2 scores for different number of clusters centers. **a** The highest VAMP2 score for the WT system corresponds to k=60. **b** The highest VAMP2 score for the WTF system corresponds to k=150. **c** The highest VAMP2 score for the XBB system corresponds to k=20. **d** The highest VAMP2 score for the XBBF system corresponds to k=30.

Figure S2

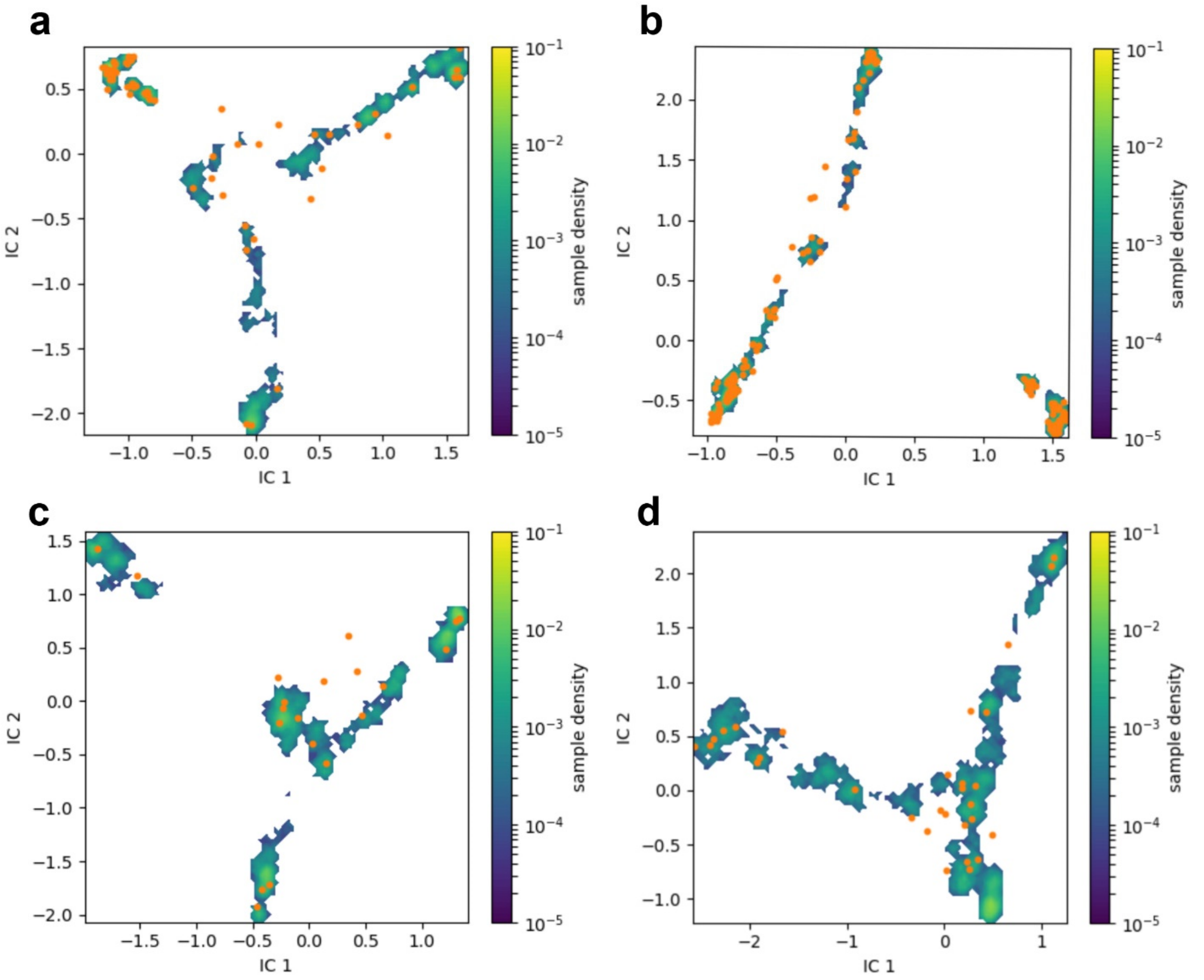

Figure S2. K-means clustering results of TICA-reduced trajectories data. **a** Clustering result of WT. **b** Clustering result of WTF. **c** Clustering result of XBB. **d** Clustering result of XBBF.

Figure S3

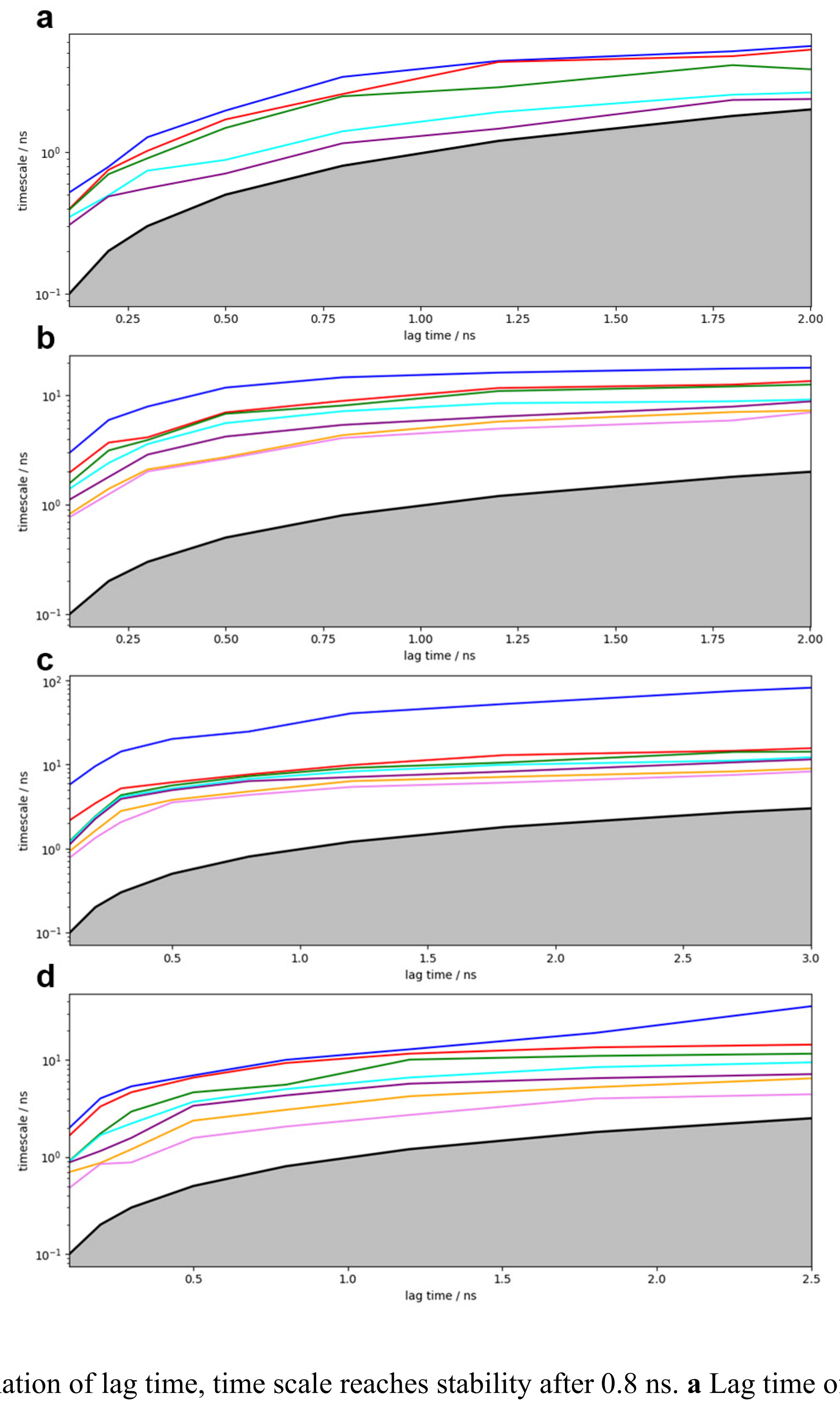

Figure S3. Determination of lag time, time scale reaches stability after 0.8 ns. **a** Lag time of WT. **b** Lag time of WTF. **c** Lag time of XBB. **d** Lag time of XBBF.

Figure S4

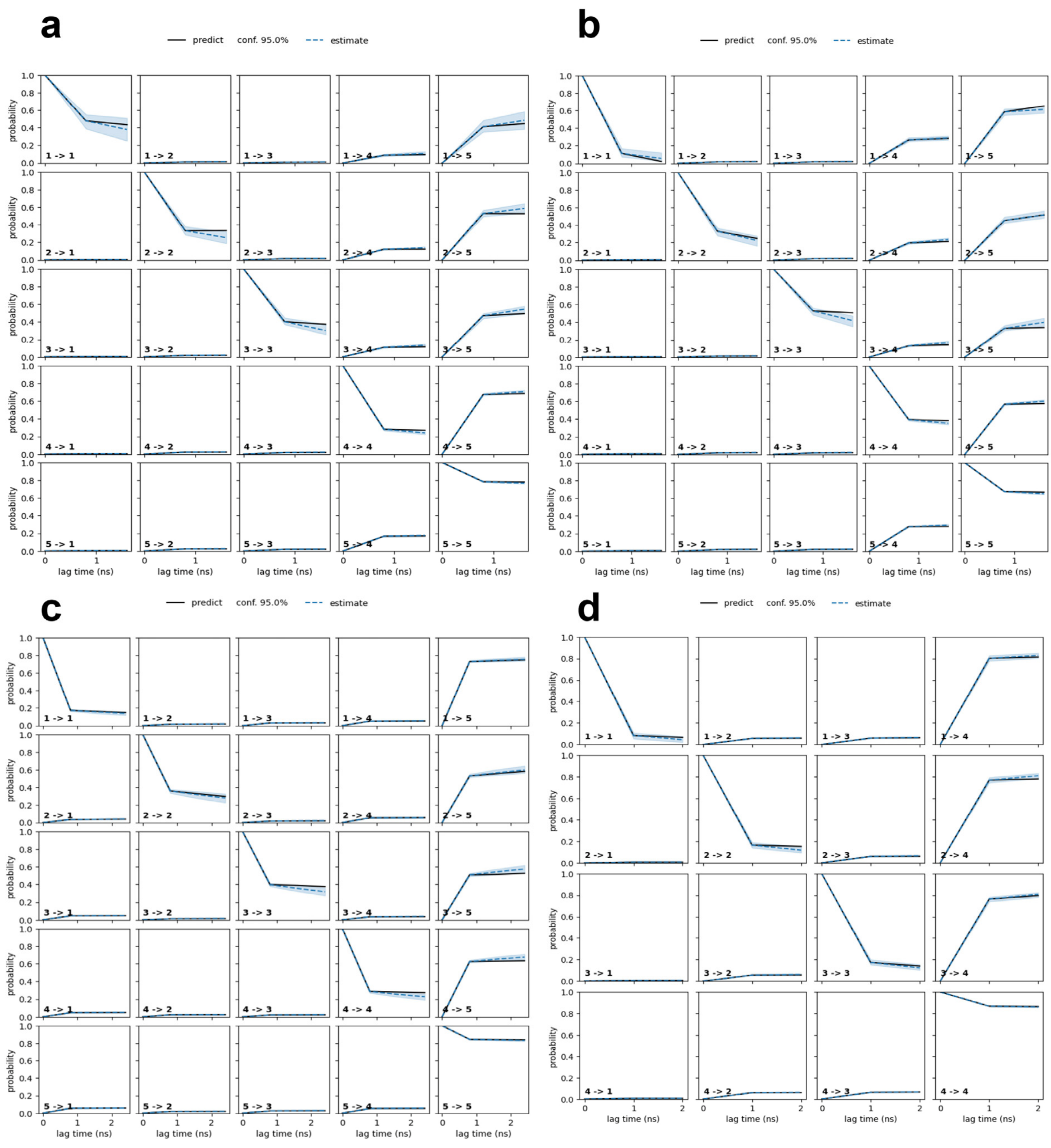

Figure S4. Results of Chapman-Kolmogorov tests for dividing systems into metastable states, a nearlyagreement were observed for all the lag time, indicating the high Markovianity of the microstates, these metastable states are reasonable choices for our MSM. **a** WT. **b** WTF. **c** XBB. **d** XBBF.
